# The Influence of Preforming Protein Coronas on the Performance of Dengue NS1 Immunoassays

**DOI:** 10.3390/pharmaceutics14112439

**Published:** 2022-11-11

**Authors:** Hom Rijal, Laura Goggin, Rachel Muriph, Jason Evans, Kimberly Hamad-Schifferli

**Affiliations:** 1 Department of Chemistry, University of Massachusetts Boston, Boston, MA 02125, USA; 2 Department of Engineering, University of Massachusetts Boston, Boston, MA 02125, USA; 3 School for the Environment, University of Massachusetts Boston, Boston, MA 02125, USA

**Keywords:** sandwich immunoassay, nanoparticles, protein corona, lateral flow assay, rapid diagnostic, dengue, nonstructural protein 1

## Abstract

The effect of preformed protein coronas on immunoassays for Dengue nonstructural protein 1 (NS1) immunoassays was investigated. The composition of the protein corona that forms around nanoparticle–antibody conjugates in human serum was characterized, and selected proteins from the corona were used for preformed coronas (human serum albumin and apolipoprotein A1). Coronas were formed and characterized by dynamic light scattering (DLS), and the nanoparticle-conjugate was probed by optical absorption spectroscopy. Immunoassays were run, and performance was quantified by analyzing the strip intensity as a function of NS1 concentration. The preformed coronas influenced the limit of detection (LOD) of the assay and the affinity for the NS1 target (*K_D_*). The resulting *K_D_* and LODs for the NP–Ab–ApoA1 immunoprobes were 0.83 nM and 1.24 nM, respectively. For the NP–Ab –HSA coronas, the test line intensity was lower by 33% at a given NS1 concentration than for the NP–Ab immunoprobes, and *K_D_* was 0.14 nM, a slightly higher affinity. Due to the relatively large error of the negative control, a meaningful LOD for the NP–Ab with HSA coronas could not be determined.

## 1. Introduction

Infectious disease diagnostics are important tools for disease containment, patient treatment and surveillance. In particular, the point of care format has been widely used, which is often in the form of lateral flow assays (LFAs) or dipstick immunoassays that target viral antigens. LFAs rely on gold nanoparticles (NPs) functionalized with an antibody specific for the target. If the antigen is present, it binds to both the NP–antibody and the antibodies immobilized at the test line, forming a sandwich immunoassay. This double-binding event accumulates NPs at the test line and, due to the strong optical absorption of NPs, results in a colorimetric signal that can be read out by eye. LFAs have been attractive as point-of-care diagnostics because they are lightweight, robust, can be operated by non-experts and produce an answer within minutes.

While the LFA format has been used for decades, one of its major limitations is its low sensitivity, as it requires enough NPs to be accumulated to generate a visible or imageable signal. Diagnostic performance is quantified by the limit of detection (LOD), the lowest detectable antigen concentration, and this quantity is important for assessing whether it can be used as a diagnostic tool in a given setting, i.e., whether the test can detect the disease biomarker at a stage of infection sufficiently early to aid in response. Rapid COVID-19 diagnostics that are read out visually have LODs ~10 s of pg/mL to ng/mL and can potentially miss the window for detection. For instance, infection with SARS-CoV-2 does not result in a secreted protein present at high concentration, so the antigen tests must be able to detect the viral proteins themselves [1]. Thus, a patient needs to build up enough viral load for the protein to be detectable, so advice from public health agencies is to take antigen tests two or three times over the span of a few days for the most accurate answer.

At a given antigen concentration, there are many factors that influence test line intensity in an immunoassay, including the concentration of the NP–antibody conjugate and the physical properties of the NP, such as particle size and shape [2]. However, another important but less-studied factor influencing immunoassay performance is protein coronas, where proteins adsorb via electrostatic, van der Waals, and hydrophobic interactions to NP surfaces to spontaneously form a “cloud” around the NPs when they are in biological fluids. LFAs are routinely run with biological fluids, such as blood, serum and saliva, and these fluids have proteins at high concentrations, so a protein corona is formed around the NP–antibody conjugate as the fluid migrates through the strip [3].

The protein corona has been well-studied, where the field now has a solid understanding of its physical properties and behavior, including its composition, protein exchange kinetics and how these change with the environment [4,5]. Approaches to leverage the unique properties of protein coronas have been investigated, where preforming the coronas can be used to influence biological outcomes. Ritz et al. examined this phenomenon with cell uptake of polystyrene NPs, where they identified proteins in the corona and how they each correlated with cell uptake. They found that preforming a pure protein corona of ApoH increased cell uptake, but coronas made of ApoA3 or ApoA4 decreased it [6]. This shows that control over protein corona composition can regulate cellular uptake of NPs and that control over corona composition can potentially enable the design of the NP “biological identity” to engineer their properties for different nanomedicine applications.

Protein coronas have been studied predominantly for cancer applications, where numerous reports have investigated the influence of coronas on chemotherapeutic delivery and targeting in vivo and how they impact interactions of NPs with specific cell receptors [7,8,9,10]. However, the influence of protein coronas is broad, and they can impact many other biological uses of NPs including LFAs. Previously, it has been shown that presence of protein coronas improves the efficacy of paper immunoassays, where they prevent false positives by diminishing non-specific adsorption [11,12]. However, this was done without the knowledge about the protein corona composition, so the effects of individual proteins was not known and there are still opportunities to engineer the protein corona to improve LFA performance.

**Figure 1:**
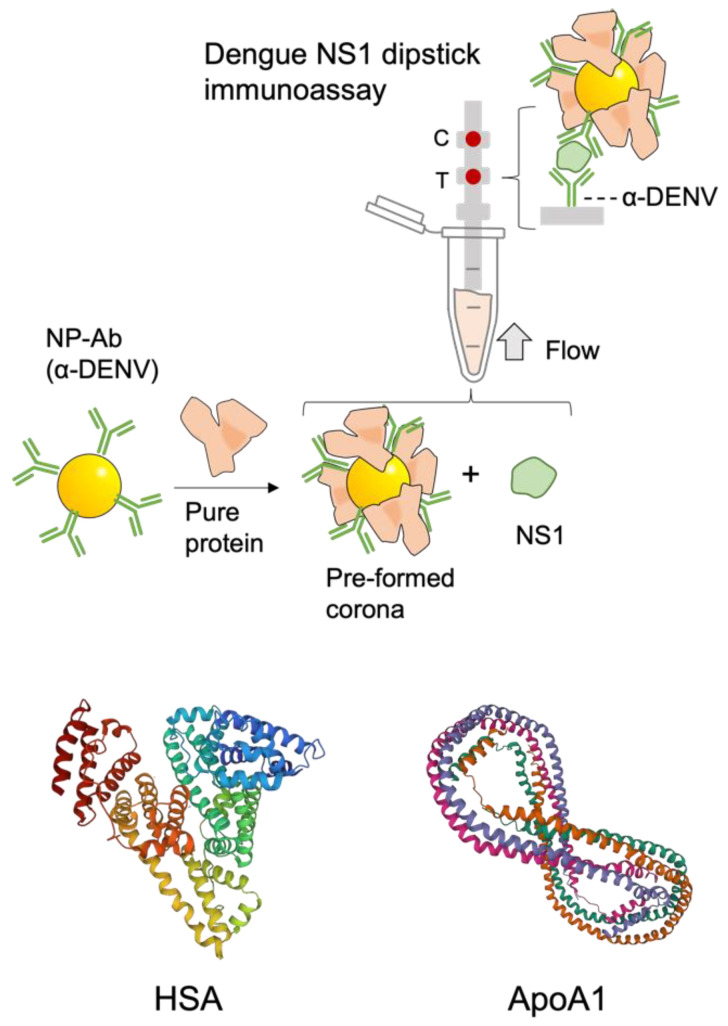
Protein coronas of pure proteins are formed around NP–Ab conjugates which are then run in dipstick immunoassays for dengue NS1. The test area of the strip (T) was spotted α–DENV and the control area (C) anti-Fc. When the target NS1 is present in the sample, t color appears at both T and C. If no NS1 is present, no color appears at C. Protein structure images [13] of Apo A1 (PDB 1AV1) from Bohani et al. [14] and HSA (PDB ID) 1AO6 from Sugio et al. [15].

Here, we examined the effect of the composition of protein coronas on dipstick immunoassays (Figure 1). We characterized the protein corona composition in an immunoassay for a dengue biomarker run in human serum (HS) using liquid chromatography tandem mass spectrometry (LC–MS/MS). We then preformed coronas from two different proteins identified in the corona, apolipoprotein A1 (Apo A1) and human serum albumin (HSA). We find that the preformed PCs influence immunoassay performance differently depending on the protein used, altering the *K_D_* and LOD values. Results could be used to improve immunoassay performance, which can impact the capability of a diagnostic for its use in infectious disease surveillance and quarantining.

Dengue virus is a flavivirus and is the fastest growing mosquito-borne disease, with up to 400 million cases diagnosed a year and 40,000 of those cases being fatal. According to the Centers for Disease Control, approximately 4 billion people are at risk for infection of dengue, a number which has been steadily increasing due to increased travel and growth of the habitat for its vectors, the mosquitoes *Aedes aegypti* and *Aedes albopictus* [16,17]. Dengue infection can progress to the more serious and fatal dengue fever, which afflicts approximately 100 million people per year. Though a vaccine is available, accessibility in more rural communities is limited and puts these individuals at greater risk for complications. Fatalities of dengue fever can be reduced with early intervention and treatment, underscoring the importance of early diagnosis and consequently the sensitivity of dengue LFAs.

Rapid diagnostics for dengue infection target the biomarker nonstructural protein 1 (NS1) because it is secreted at high concentrations into the blood stream starting soon after the infection onset [18]. Using immunoassays for NS1 of dengue serotype 2, we investigated the effect of preforming protein coronas around the NP–anti-dengue antibody conjugates.

## 2. Materials and Methods

### 2.1. Gold NP Synthesis

49.5 g Milli-Q water was weighed into a glass bottle, and 0.5 mL of a 10-mg/mL solution of HAuCl_4_ (≥99.9% trace metals basis, Sigma) was added to it, after which the mixture was placed in a water bath, stirred and left to fully equilibrate for 15 min. The lid was carefully opened, and 0.9 mL of 10-mg/mL sodium citrate (≥99.0%, tribasic sodium citrate, Sigma) was added under vigorous stirring. The lid was replaced, and the solution was left to react for ~15 min. During this time, the solution changed color from pale yellow to almost black to wine red as NPs formed. 0.1 mg of bis(p–sulfonatophenyl)phenylphosphine dihydrate dipotassium salt (Sigma Aldrich, St. Louis, MO, USA, 97%, BPS) was added to the NPs as a stabilizer.

### 2.2. Immunoprobe (NP–Ab Conjugate) Preparation

The gold NPs were concentrated by centrifugation at 2348× *g* for 12 min, and the pellet was resuspended in 300 μL of Milli-Q water, 100 μL of 40 mM HEPES; 10 μL of the antibody at 1 mg/mL (anti-Dengue NS1 polyclonal antibodies, Sino Biological) was added. The solution was left on the shaker for 1 h at room temperature, and 10 μL of PEG (MW 5000, 0.1 mM) backfill was added and left for 15 min on the shaker at room temperature to cover the unpassivated areas on the NP surface, which helps to avoid signal interference. The nanoparticles were centrifuged for 12 min at 2348× *g* to separate the excess antibody and PEG. Then the solution was resuspended to a volume of 50 μL in PBS buffer (Supplementary Material, Figure S2).

### 2.3. Running Immunoassays

Nitrocellulose strips (Unistart CN140 from Sartorius) were laser cut to the desired shape. Anti-dengue antibodies were immobilized onto the nitrocellulose at the test areas by spotting 0.3 μL of solution and then were left to completely dry. Anti-Fc IgG antibodies were immobilized in the control area as verification that fluid flow and the immobilized and conjugated antibodies worked correctly.

An absorbent pad attached to the top of the strip served as a fluid reservoir. The bottom of the strips was placed in a nanoparticle solution with 20 μL of human serum with a dengue NS1 protein. The fluid was allowed to wick up the strip by capillary action toward the absorbent pad for ~15–30 min (GB003 Gel Blot paper, Sigma–Aldrich). Immunoassays were run at room temperature (Supplementary Material, Figure S3).

### 2.4. Image Analysis

After completely drying up the test strips, the strips were affixed to paper and scanned on a desktop scanner [19]. The mean gray value of the measurement tool was used to obtain the grayscale values’ use of the test area by using ImageJ [20]. The area of the rectangle used was fixed to be within the particle signal and kept constant for each image analysis. The background grayscale value was subtracted from the signals, and each strip had a separate background measurement located just below or above the test area. Antigen titrations were fitted with a single Langmuir curve using Matlab to obtain effective *K_D_* and LOD values.

### 2.5. LOD *K_D_* Analysis

Test line intensities as a function of NS1 concentration were fit to Langmuir isotherms in Matlab, according to equations in previous reports [11,21]. LOD was defined as the concentration at which the strip intensity was 3 × SD of negative control above the baseline intensity ([NS1] = 0 nM).

### 2.6. Protein Corona Formation and Analysis

#### 2.6.1. Protein Corona Formation

The conjugated gold NP pellet was suspended in 1 mL of either 5% (*v*/*v*) of HS (Sigma Aldrich), HSA (97%, Sigma) or Apolipoprotein A1 (>98% purity, EMD Millipore, St. Louis, MO, USA). Phosphate Buffer (PB) was added to yield a final concentration of 0.l mM (Boston Bioproducts, Milford, MA, USA) in MilliQ water. Once all the reagents were added, the solution was vortexed and left to incubate overnight at 37 °C. After the overnight incubation, it was then centrifuged at 5000× *g* for 12 min. The supernatant was discarded, and the pellet was resuspended in 25 µL of Milli-Q water.

#### 2.6.2. Protein Corona Detachment from AuNP

To remove the corona proteins from the NP–Ab immunoprobes, we used a procedure from the literature and our prior work that was found to be sufficient for mass spectrometry analysis [22,23]. NP–Ab immunoprobes were centrifuged at 15,000× *g* for 75 min and resuspended in 1 mL of 0.05% Tween–20–PBS mixture. It was then centrifuged again at the same speed for 45 min and resuspended in the Tween PBS mixture. At the third spin, it was resuspended in 8.0 µL of NuPAGE 4X LDS buffer and 4.0 µL of 500 mM DTT. It was placed in a 70 °C water bath for 60 min and then was centrifuged at 15,000× *g* for 15 min, and the supernatant was separated and placed in a new tube. The aliquot was used for SDS–PAGE analysis and Mass Spectrometry analysis.

#### 2.6.3. Tryptic Digestion of the Detached Protein for Mass Spectrometry Analysis

500 µL of 10% (*w*/*v*) Trichloroacetic acid (TCA) in acetone was added to the supernatant and left in −80 °C overnight. Afterward, it was spun at 15,000× *g* for 35 min and resuspended in 500 µL of 0.03% (*w*/*v*) sodium deoxycholate and 100 µL of 72% TCA. The sample mixture was then vortexed and incubated on ice for 60 min. Following this incubation, it was centrifuged at 18,000× *g* for 15 min, resuspended in 1 mL of cold acetone and stored in −80 °C for 60 min. The sample was centrifuged again using the previous setting, and the resulting pellet was air-dried in a concentrator (Savant Speed Vac SC 100) for 15 min with the settings set on high.

Sample preparation for analysis via mass spectrometry was adapted from a commercial protocol (Thermo Fisher Scientific: Mass Spectrometry-Grade Endoproteinases). The air-dried protein pellet was resuspended in 30 µL Optima Water (Fisher Chemical, Fair Lawn, NJ, USA), 10 µL of 500 mM digestion buffer (ammonium bicarbonate, Fisher Chemical, Fair Lawn, NJ, USA) and 2 µL of 500 mM dithioerythritol (DTT), 99+% (Acros Organics). The proteins were reduced at 60 °C for 45 min. The samples were cooled to room temperature for 10 min before the addition of 3.5 µL of 500 mM IAA (Acros Organics). The samples were alkylated at room temperature for 20 min in the dark. The sample volumes were adjusted to 50 µL with Optima water. The samples were then digested overnight at 37 °C via the addition of 2.5 µL of 1 mg/mL Trypsin/Lys–C protease cocktail (mass spec grade, Promega). An Orbitrap Fusion Lumos mass spectrometer (Thermo Fisher, San Jose, CA, USA), coupled online to an EASY–nLC 1200 (Thermo Fisher, San Jose, CA, USA), was used to perform the RP–nLC/ESI–MS analysis. A detailed operational description is provided in the Supplementary Material.

### 2.7. MS Data Analysis with Proteome Discoverer 2.4

Proteome Discoverer 2.4.1.15 software (Thermo Fisher, San Jose, CA, USA) was used to analyze the raw data files. Spectra were selected using MS1 precursors between 350–5000 Da. The data were run versus the Homo sapiens UniProt FASTA file (Modified 2 March 2022). The data were searched using Sequest HT with the following parameters: Trypsin (Full), a maximum of two missed cleavage sites, peptide lengths between 6–150 amino acids, a precursor mass tolerance of 10 ppm, fragment mass tolerance 0.6 Da, dynamic modifications for oxidation of methionine (+15.995 Da) as well as an acetylation of the protein terminus (+42.011 Da); a static modification was set for carbamidomethyl on cysteine (+57.021 Da). The Percolator node was used to validate the results using a decoy database search with a strict target False Discovery Rate (FDR) of 0.01 (high confidence) and a relaxed target FDR of 0.05 (medium confidence) with validation based on q-value. Peptides and proteins were quantified with the Precursor Ions Quantifier node in conjunction with the Minora Feature Detector node to obtain abundance values per peptide. All peptides were used, and protein groups were considered for peptide uniqueness. The maximum allowed fold charge was 100 and the top three peptides were used for area calculations.

## 3. Results

### 3.1. Synthesizing NP–Antibody Conjugates

**Figure 2:**
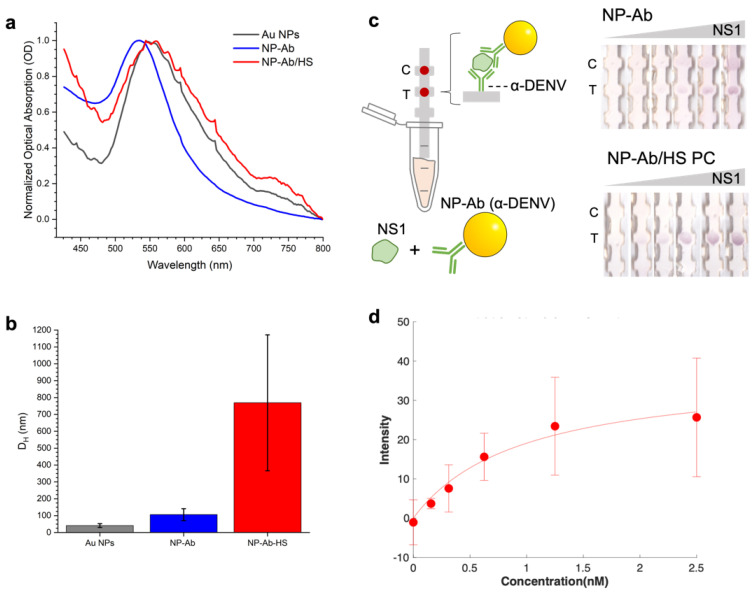
(**a**) Optical absorption of Au NPs (gray), NPs conjugated to anti–Dengue NS1 antibodies, NP–Ab (blue) and NP–Ab with preformed coronas from human serum (NP–Ab/HS, red). (**b**) DLS of Au NPs, NP–Ab and NP–Ab/HS. Error bars indicate error from ten measurements. (**c**) Image of immunoassay strips run with dengue NS1 and (**d**) test area intensities as a function of NS1 concentration (red circles) and fit to obtain the LOD and *K_D_* values (line). Error bars due to immunoassays run in triplicate.

NP–antibody conjugates for the assay were synthesized. Au NPs were synthesized in aqueous solution using sodium citrate reduction of HAuCl_4_, which results in spherical particles [24,25], and were passivated with bis(p–sulfonatophenyl)phenylphosphine dihydrate dipotassium salt (97%, BPS) to improve stability in biological fluids [26]. Optical absorption spectroscopy revealed a SPR peak at 546 nm (gray, Figure 2a), which exhibited a narrow absorbance, suggesting minimal NP aggregation. Dynamic Light Scattering (DLS) of the NPs obtained a *D_H_* of 41.2 ± 11 nm (gray, Figure 2b). The NPs were conjugated to anti-dengue 2 NS1 by adsorption, where the NPs were incubated in solution with the antibody at a 1:150 NP:antibody molar ratio, followed by a backfill with thiolated PEG to reduce non-specific adsorption by passivating free surface. Free antibody and PEG were removed by spin centrifugation, leaving the NP–antibody in the pellet. DLS of the NP–Ab showed an increase in *D_H_* to 106.1 ± 35.3 nm (blue, Figure 2b), and the SPR peak did not exhibit significant broadening (blue, Figure 2b), which confirmed successful conjugation of the anti-Dengue antibodies to the Au NPs with minimal NP aggregation.

Dengue NS1 immunoassays were run using the NP–Ab conjugates in dipstick assays (Figure 2c). Nitrocellulose strips had anti-dengue antibody immobilized at the test area and the anti-Fc antibody on the control area. A solution of the NP–anti-dengue immunoprobe, human serum, Tween/sucrose running buffer and a variable amount of the NS1 target was put into a centrifuge tube, and strips were run by immersing the bottom of them in the solution, which wicked up the strip towards the absorbent pad.

When the NS1 target was present, a red color appeared at the test line (right most strip, position T, Figure 2c). This indicated that NPs were accumulated at that location and that the NS1 could successfully form a sandwich between the immobilized antibody and the NP–Antibody conjugate. When no NS1 was present (leftmost strip, position T), no color appeared at the test line, showing that no non-specific adsorption occurred. The control line still exhibited a signal (leftmost strip, position C), confirming that proper flow occurred.

**Figure 3:**
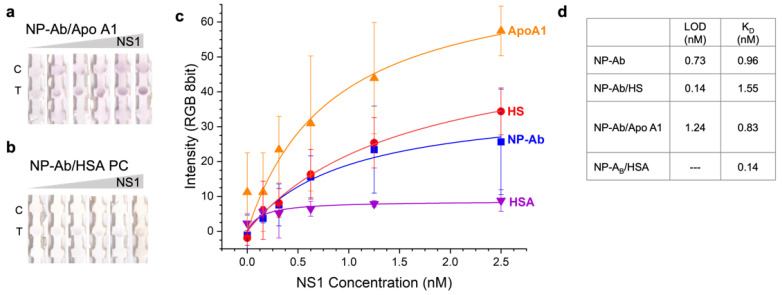
Dengue NS1 immunoassays run with preformed coronas of (**a**) Apo A1 and (**b**) HSA around the NP–Ab conjugates. (**c**) Test line intensities as a function of NS1 concentration for NP–Ab (blue) with different protein coronas of ApoA1 (orange), HSA (purple) and human serum (HS, red). Error bars are from three independent measurements. (**d**) LOD and *K_D_* values.

To evaluate the immunoassay performance, we quantified the test area intensities by image analysis with ImageJ as the area’s 8 bit RGB intensity above background [12]. We measured the test line intensity as a function of NS1 concentration (Figure 2d and Figure S1.). The intensity increased with NS1 concentration, showing that increasing the NS1 concentration accumulates more NP–Ab/target. The concentration dependence was fit [11] to obtain an effective binding affinity, *K_D_*, and a limit of detection (LOD). A *K_D_* of 0.96 nM was obtained, and the LOD was 0.73 nM (Figure 3d).

### 3.2. Protein Corona Characterization

Because the immunoassay is run in HS, we characterized the corona that formed around the NP–Ab in HS. Protein coronas were formed by incubating the NP–Ab in HS at 37 °C for 24 h, and then free proteins were purified from the NP–Ab–HS by spin centrifugation. The NP–anti-dengue conjugates with HS coronas (NP–Ab–HS) exhibited an increase in *D_H_* to 800 nm as evidenced by DLS (red, Figure 2b), and the SPR red shifted to ~550 nm and slightly broadened (red, Figure 2a), confirming corona formation. This can be attributed to a change in the local index of refraction in combination with moderate aggregation of the NPs upon corona formation. Other approaches to characterize coronas in the literature include high resolution microscopy, such as STED microscopy [27]. NP–Ab–HS were still functional in immunoassays for dengue NS1. At a given concentration of NS1, the test line still appeared, and there was no signal at the test line when NS1 was not present, demonstrating that the formation of the HS protein corona did not impact the immunoprobe function.

We used LC–MS/MS to identify the proteins in the corona that formed around the NP–Ab in HS. The protein corona was isolated from the NP–antibody conjugates by heating and detergents [23], and NPs were separated from the corona proteins by spin centrifugation. Corona proteins were prepared for MS by precipitation with acetone and then digested with trypsin. After keratin subtraction of the MS identifications, the most abundant proteins were identified (Table 1 and Table S1).

Due to its abundance in human serum and ability to form coronas around NPs, human serum albumin (HSA) was chosen to create preformed coronas in order to investigate the effect of these proteins on lateral flow immunoassays. HSA is a 69.3 kDa protein secreted by the liver and is the most abundant protein found in human serum [28]. Consisting of mainly three large domains, it mainly binds with long chain fatty acids to allow for their transport through the bloodstream to various parts of the body [29]. Due to its natural tendency to form interactions with small hydrophobic molecules but its tendency to change configurations easily under pH conditions [30], HSA is viewed as a soft corona protein for many NPs where it binds relatively weakly to the NP surface and is more prone to exchange [22].

We also selected apolipoprotein A–1 (Apo A1), a 30.8 kDa protein that was the fourth most abundant in the corona. Apo A1 is synthesized in the liver with an approximate concentration of 100–15 mg/dL in human plasma [31] and is vital for the delivery of specific lipids to their target cells and organ systems. Apo A1 is the main component of High Density Lipoprotein (HDL), a key regulator of cholesterol levels within the human body [32]. Its main structural feature is an amphiphilic helix, which gives it the ability to form tight coronas around a variety of NPs and is in dimeric form [33].

### 3.3. Effect of Preformed Coronas of Pure Proteins

**Figure 4:**
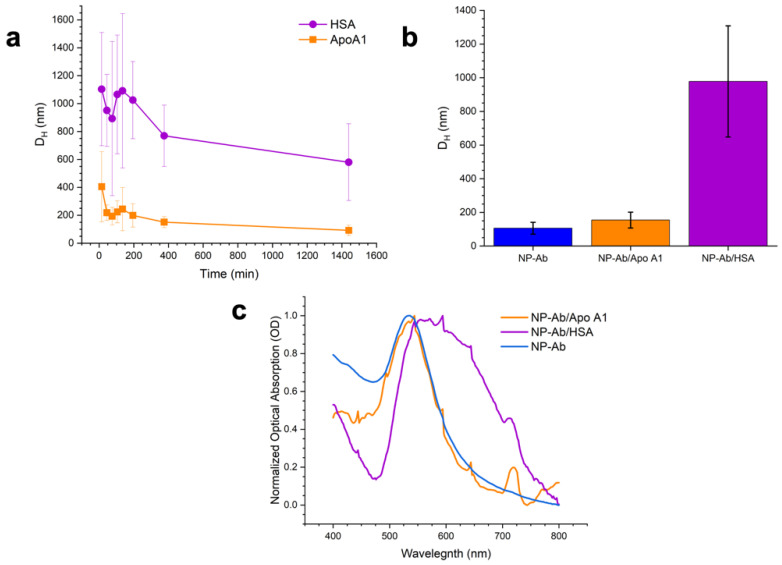
(**a**) DLS with time for NP–Ab incubated with HSA (purple) and Apo–A1 (orange). (**b**) DLS of final preformed coronas (after 24 h) for Apo A1 and HSA compared to the NP–Ab with no corona (blue). (**c**) Optical absorption spectra of NP–Ab/Apo A1 (orange), NP–Ab/HSA (purple) and NP–ab (blue).

We incubated the NP–Ab conjugates with each of the pure proteins and measured *D_H_* with time by DLS to verify corona formation (Figure 4a). The *D_H_* initially increased and then decreased over time before stabilizing at longer times (24 h). Studies of the kinetics of corona formation [5,34] have shown that initially a corona is formed rapidly upon introduction of the NP into HS or other media, often within milliseconds, which is then followed by an exchange over a longer time period of hours where proteins of higher affinity exchange with proteins in the corona [22]. The observed exchange behavior could result in exchange of the anti-dengue antibodies on the NPs with the HSA or Apo A1 due to differences in affinity for the NP surface [35,36]. Based on the time-dependent DLS results, we determined that >24 h was sufficient for Apo A1 and HSA to form stable coronas around the NP–Ab conjugates (Figure 4a,b). The SPR of NP–Ab/Apo A1 did not show a significant shift relative to NP–Ab (orange, Figure 3c), but the NP–Ab/HSA did, suggesting that the HSA corona compromised NP stability somewhat.

We then used the NP–Ab conjugates with the preformed coronas in immunoassays and evaluated immunoassay performance. For coronas of Apo A1 (Figure 3a), the immunoprobes resulted in a positive test signal when NS1 was present and no test signal when NS1 was not, demonstrating specificity for the target and that the corona did not impact its ability to bind to the target. The concentration dependence (Figure 3c) was similar to the immunoprobes with no preformed coronas and HS coronas, and the test line intensity was not significantly compromised with the Apo A1 coronas. The resulting *K_D_* for the NP–Ab–ApoA1 immunoprobes was 0.83 nM, (Figure 3d) and LOD 1.24 nM (Figure 3d), so the Apo A1 corona decreased the test’s affinity and sensitivity somewhat.

For NP–Ab conjugates with coronas of HSA, the immunoprobes were still functional in an immunoassay (Figure 3b). However, the test line intensity was much lower than for the NP–Ab immunoprobes themselves, as low as 33% at a given NS1 concentration. This could be due to the HSA corona possibly decreasing the Ab coverage on the NP by protein exchange or inhibiting the ability of the NP–Ab to bind to the target. The resulting *K_D_* was 0.14 nM, a slightly higher affinity (Figure 3d). Due to the relatively large error of the negative control, the fit was unable to determine a meaningful LOD for the NP–Ab with HSA coronas.

## 4. Conclusions

In conclusion, we find that the composition of the protein corona that forms in immunoassays can influence its performance. These preformed coronas can impact the LOD of the assay and the binding affinity of the NP–Ab for its antigen target. Depending on the protein, the preformed coronas had a different impact on the assay behavior. HSA coronas lowered the test intensity, most likely due to exchange of the anti-dengue antibodies on the NP surface. On the other hand, Apo A1 did not impact intensity but increased the assay LOD slightly. Even though these changes appear to be small and only the HSA corona results in a statistically significant difference, the coronas still impact the performance of the assay, which can affect its ultimate use as a diagnostic test. While only two pure proteins were studied here for preforming the corona, there are many other species in HS that could impact test performance, and future work includes investigation of the other proteins present in the corona. These results show the significance of the protein corona in immunoassays and could be used to help engineer LFAs with improved properties, in combination with typical routes to optimize immunoassay properties, such as blocking agents, engineering the immunoassay strip materials and dimensions, nanoparticle physical properties and choice of antibodies with higher affinity. This could be incorporated into the workflow where protein coronas are formed before NP–Ab conjugates are incorporated into the test, as a formulation that impacts its binding properties and ultimately tests performance.

## Figures and Tables

**Table 1:** Top 20 protein identifications from protein coronas formed in human serum derived from LC–MS/MS data.

Abundance	Protein	% Abundance
1	Albumin	52.1%
2	Alpha-1-antitrypsin	7.5%
3	Apolipoprotein A-I	3.9%
4	Immunoglobulin heavy constant gamma 1	3.8%
5	Immunoglobulin gamma-1 heavy chain	3.8%
6	Methylguanosine phosphate-specific 5′-nucleotidase	2.8%
7	Immunoglobulin kappa constant	2.5%
8	Alpha-1-acid glycoprotein 1	2.0%
9	Serotransferrin	1.7%
10	Carabin	1.4%
11	Alpha-1-antichymotrypsin	1.3%
12	Glial fibrillary acidic protein	0.9%
13	Haptoglobin	0.9%
14	Alpha-2-macroglobulin	0.8%
15	Complement C3	0.6%
16	Transthyretin	0.6%
17	Immunoglobulin heavy constant alpha 1	0.6%
18	Apolipoprotein A-II	0.6%
19	Hemopexin	0.5%
20	Alpha-1-acid glycoprotein 2	0.5%

## Data Availability

Not applicable.

## Supplementary Materials

The following supporting information can be downloaded at: https://www.mdpi.com/article/10.3390/pharmaceutics14112439/s1, Figure S1: Strip images; Figure S2: Procedure for NP–antibody conjugation; Figure S3: Procedure for running dipstick immunoassays; Table S1: Top 50 protein identifications from LC–MS/MS data; RP–nLC/ESI–MS conditions.
