# Supplementary material for: The Influence of Preforming Protein Coronas on the Performance of Dengue NS1 Immunoassays
Source: Pharmaceutics. 2022 Nov 11;14(11):2439. doi: 10.3390/pharmaceutics14112439 (PMC9694804; doi:10.3390/pharmaceutics14112439)
Supplement: Supplementary file 1 [file pharmaceutics-14-02439-s001.zip › pharmaceutics-1993626-supplementary.pdf]

Supporting Information

The Influence of Preforming Protein Coronas on the Performance of Dengue NS1 Immunoassays

[1]Hom Rijal, [1]Laura Goggin, [1]Rachel Muriph, [1]Jason Evans*, [2,3]Kimberly Hamad-Schifferli*

[1]Department of Chemistry, University of Massachusetts Boston, Boston, MA 02125, USA

[2]Department of Engineering, University of Massachusetts Boston, Boston, MA 02125, USA

[3]School for the Environment, University of Massachusetts Boston, Boston, MA 02125, USA

*Contact information: jason.evans@umb.edu (J.E.); kim.hamad@umb.edu (K.H.-S.)

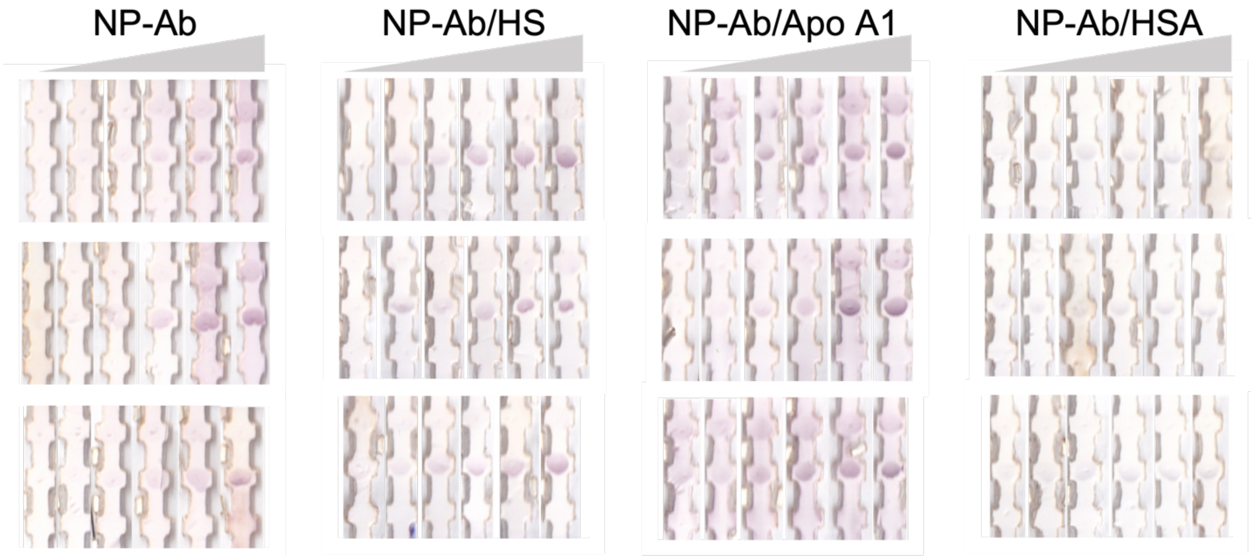

Figure S1. Images of strips of NP-Ab with different preformed coronas (no corona, HS, ApoA1, and HSA) run with increasing amounts of Dengue NS1. NS1 concentrations are 0 nM, 0.156 nM, 0.312 nM, 0.625 nM, 1.25 nM, and 2.5 nM.

Table S1. Top 50 protein identifications from protein coronas formed in human serum derived from LC-MS/MS data.

	**Protein**	**Biological Process**
1	Albumin	stress response;other biological processes
2	Alpha-1-antitrypsin	stress response;other biological processes
3	Immunoglobulin kappa constant	cell organization and biogenesis;stress response;transport;signal transduction;other biological processes
4	Apolipoprotein A-I	cell cycle OR cell proliferation;cell organization and biogenesis;protein metabolism;other metabolic processes;transport;developmental processes;signal transduction;other biological processes
5	Immunoglobulin kappa light chain	other biological processes
6	Complement C3	cell-cell signaling;cell organization and biogenesis;protein metabolism;other metabolic processes;stress response;developmental processes;signal transduction;other biological processes
7	Serotransferrin	cell organization and biogenesis;stress response;transport;developmental processes;signal transduction;other biological processes
8	Immunoglobulin heavy constant gamma 1	cell organization and biogenesis;stress response;transport;signal transduction;other biological processes
9	Immunoglobulin gamma-1 heavy chain	other biological processes
10	Alpha-2-macroglobulin	stress response;developmental processes;other biological processes
11	Immunoglobulin lambda constant 2	cell organization and biogenesis;stress response;transport;signal transduction;other biological processes
12	Immunoglobulin lambda constant 3	cell organization and biogenesis;stress response;transport;signal transduction;other biological processes
13	Haptoglobin	protein metabolism;other metabolic processes;stress response;other biological processes
14	Immunoglobulin lambda-1 light chain	other biological processes
15	Immunoglobulin lambda-like polypeptide 5	cell organization and biogenesis;stress response;transport;signal transduction;other biological processes
16	Immunoglobulin lambda constant 1	cell organization and biogenesis;stress response;transport;signal transduction;other biological processes
17	Alpha-1-antichymotrypsin	stress response;other biological processes
18	Immunoglobulin lambda constant 7	cell organization and biogenesis;stress response;transport;signal transduction;other biological processes

19	Immunoglobulin lambda constant 6	cell organization and biogenesis;stress response;transport;signal transduction;other biological processes
20	Alpha-1-acid glycoprotein 1	stress response;other biological processes
21	Transthyretin	other metabolic processes;transport;signal transduction
22	Desmoplakin	cell adhesion;cell organization and biogenesis;protein metabolism;other metabolic processes;stress response;developmental processes;other biological processes
23	Immunoglobulin heavy constant gamma 4	cell organization and biogenesis;stress response;transport;signal transduction;other biological processes
24	Alpha-1-acid glycoprotein 2	stress response;other biological processes
25	Apolipoprotein A-II	cell organization and biogenesis;protein metabolism;other metabolic processes;stress response;transport;other biological processes
26	Immunoglobulin heavy constant gamma 3	cell organization and biogenesis;stress response;transport;signal transduction;other biological processes
27	Immunoglobulin heavy constant alpha 1	cell organization and biogenesis;stress response;transport;signal transduction;other biological processes
28	Immunoglobulin heavy constant gamma 2	cell organization and biogenesis;stress response;transport;signal transduction;other biological processes
29	9 Actin, cytoplasmic 1 "	cell organization and biogenesis;protein metabolism;other metabolic processes;stress response;transport;developmental processes;other biological processes
30	1 Actin, cytoplasmic 2 "	cell organization and biogenesis;stress response;transport;developmental processes;other biological processes
31	Alpha-enolase	other metabolic processes;other biological processes
32	Haptoglobin-related protein	protein metabolism;other metabolic processes;stress response;other biological processes
33	Clusterin	cell cycle OR cell proliferation;cell organization and biogenesis;protein metabolism;other metabolic processes;stress response;transport;developmental processes;signal transduction;other biological processes
34	Protein S100-A9	cell adhesion;cell-cell signaling;protein metabolism;other metabolic processes;stress response;developmental processes;other biological processes
35	Junction plakoglobin	cell adhesion;cell organization and biogenesis;other biological processes
36	Desmoglein-1	cell adhesion;cell organization and biogenesis;other biological processes
37	Hemopexin	protein metabolism;other metabolic processes;transport;other biological processes
38	Immunoglobulin kappa variable 1D-33	other biological processes

39	Protein S100-A7	stress response;developmental processes;other biological processes
40	Prothrombin	protein metabolism;other metabolic processes;stress response;signal transduction;other biological processes
41	Glial fibrillary acidic protein	cell organization and biogenesis;other metabolic processes;stress response;transport;developmental processes;other biological processes
42	Fructose-bisphosphate aldolase A	cell organization and biogenesis;other metabolic processes;other biological processes
43	POTE ankyrin domain family member E	
44	Antithrombin-III	stress response;other biological processes
45	Alpha-1B-glycoprotein	
46	Plasma protease C1 inhibitor	stress response;other biological processes
47	Alpha-2-HS-glycoprotein	stress response;transport;developmental processes;other biological processes
48	Complement C4-B	stress response;other biological processes
49	Complement C4-A	stress response;other biological processes
50	B-cell receptor CD22	cell adhesion;other biological processes

RP-nLC/ESI-MS conditions

The nLC system was operated at a flow rate of 300 nL/min using the following gradient: 0-4 min 0-8% B, 4-124 min 8-30 % B, 124-139 min 30-90 % B, 139-149 min 90 % B, 149-169 min at 90-0 % B, and 169-180 min 0 % B for column re-equilibration. Mobile phase solvents were purchased from Fisher Chemical (Fair Lawn, NJ, USA) and were Optima LC/MS Grade. Mobile phase A was 96.1:3.9 0.1% FA in water/0.1 % FA in ACN. Mobile phase B was 80.0:20.0 0.1 % FA in water/0.1% FA in ACN. The samples were first desalted on a Thermo Fisher Scientific Acclaim PepMap 100 C18 HPLC column (3 μm particle size, 75 μm x 2 cm, 100 Å) prior to separation on a Thermo Fisher Scientific PepMap RSLC C18 EASY-Spray Column (3 μm particle size, 75 μm x 15 cm, 100 Å).

The Orbitrap Fusion Lumos mass spectrometer was operated in the peptide mode. The global settings were as follows: ion source type NSI, positive voltage of 1900 V, and an Ion Transfer Tube Temp of 275 °C. Ions for the MS scans were detected in the Orbitrap with a resolution of 120,000. The mass range was normal, quadrupole isolation was checked, and the scan range was set to 400-1600 m/z. The RF lens was set to 30 % and the AGC target and maximum injection time were 2.0e5 and 100 ms, respectively. One microscan per scan was taken.

The following filters were utilized: MIPS, Intensity, Charge State, and Dynamic Exclusion. The MIPS filter was set to peptide. The intensity filter was set to an intensity threshold of 8.0e3. The charge state filter was set to include charge states 2-6. A dynamic

exclusion filter was configured to exclude ions after 1 time for a duration of 30 seconds with a ± 10 ppm mass tolerance. Isotopes were excluded. Following the dynamic exclusion filter, the method branched into two ddMS^2 legs with respective charge state filters. CID was collected on ions with a charge state between 2 and 3 while EThcD was collected on ions with a charge state of 3 to 6.

The first leg consisted of the charge state filter set to include charge states 2-3 and was applied before the ddMS^2 IT CID node. Ions for the ddMS^2 CID were isolated in the quadrupole with a with an isolation window of 1.2 m/z. Ions were fragmented via CID with a fixed collision energy of 35% and an activation time of 10 ms. The Q parameter for the CID activation was set 0.25. The ions were detected in the Ion Trap with an Auto: m/z Normal scan range and a rapid scan rate. The first mass was set to 100 m/z. The AGC target and maximum injection time were set to 2.0e4 and 50 ms. One microscan per scan was taken.

The second leg consisted of the charge state filter set to include charge states 3-6 and was applied before the ddMS^2 IT EThcD node. Ions for the ddMS^2 EThcD were isolated in the quadrupole with an isolation window of 1.2 m/z. Ions were fragmented via ETD using calibrated charge dependent EtD parameters and ETD supplemental activation. EThcD was used with a collision energy of 25%. The ions were detected in the Ion Trap with an Auto: m/z Normal scan range and a rapid scan rate. The first mass was set to 120 m/z. The AGC target and maximum injection time were set to 2.0e4 and 50 ms. One microscan per scan was taken. The cycle time for the data-dependent acquisition was set to 3 sec.

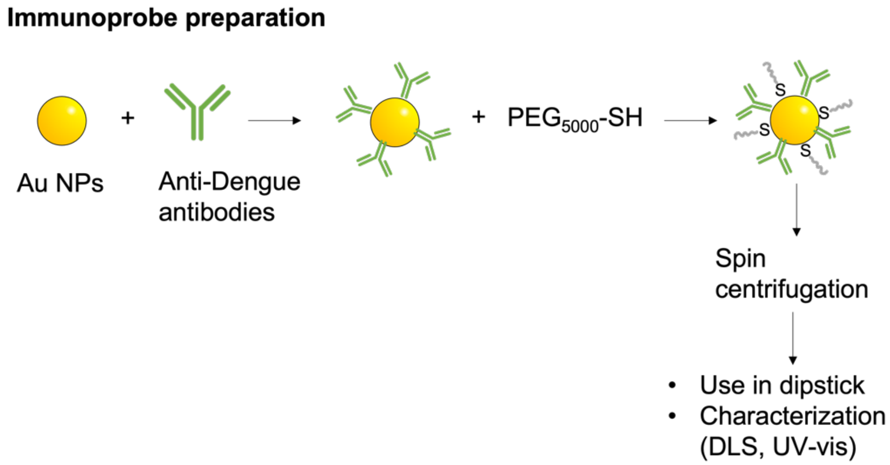

Figure S2. Procedure for NP-antibody conjugation (immunoprobe preparation).

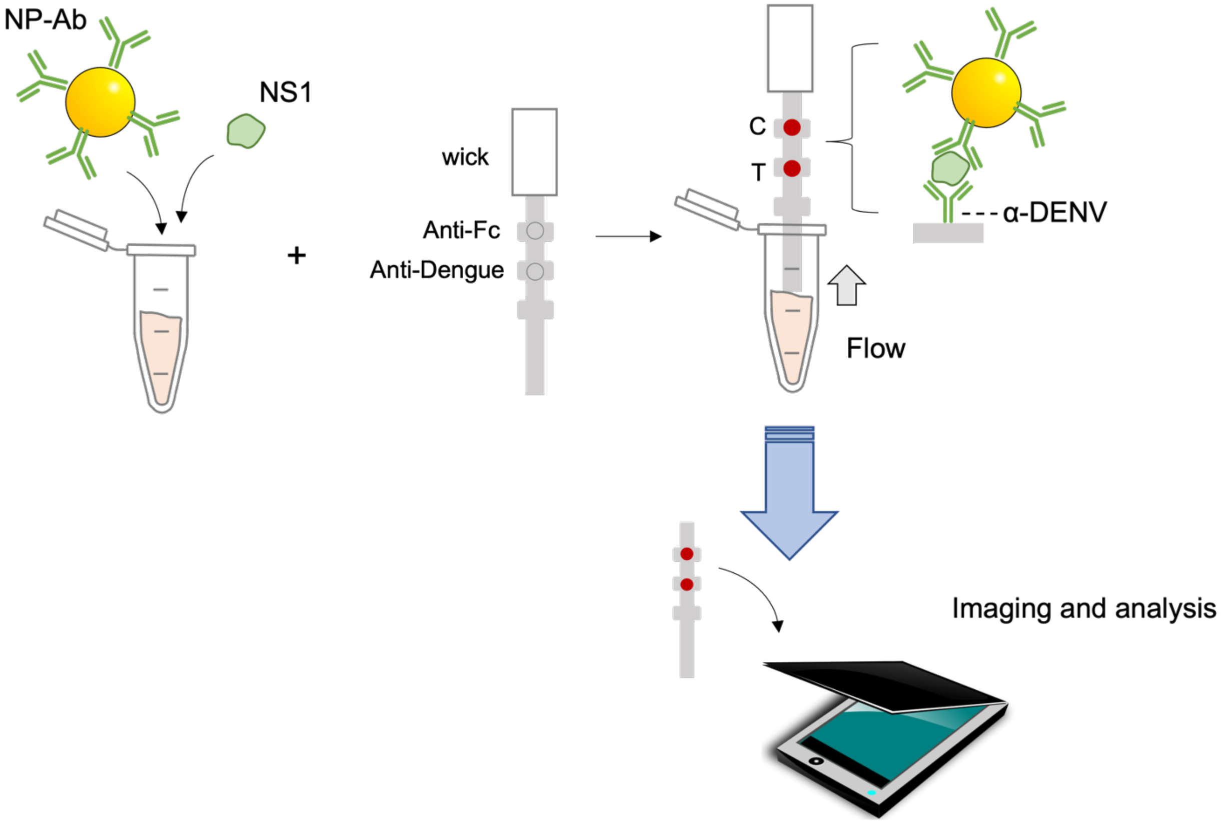

Figure S3. Procedure for running a dipstick assay as described in Methods.
